# Supplementary material for: Transcriptomic Characterization of Terminal Complement Complex-bound Cells in Human Choroid Using Single-Cell RNA Sequencing
Source: bioRxiv. 2026 Sep 10:2026.09.05.749603. Preprint. [Version 1] doi: 10.64898/2026.09.05.749603 (PMC13622589; doi:10.64898/2026.09.05.749603)
Supplement: Supplementary file 1 [file media-1.pdf]

1. Supplemental Figures

Supplemental Table 1:
Single Cell RNA Sequencing Antibodies:

BioLegend Part No.	Target	Isotype	Conjugation	Labeling Concentration	Barcode Sequence
343539	CD34	Mouse IgG1 κ	TotalSeq-B0054	1 µg/100 µL	GCAGAAATCTCCCTT
304066	CD45	Mouse IgG1 κ	TotalSeq-B0391	1 µg/100 µL	TGCAATTACCCGGAT
311321	CD55	Mouse IgG1 κ	TotalSeq-B0383	1 µg/100 µL	GCTCATTACCCATTA
304713	CD59	Mouse IgG2a κ	TotalSeq-B0361	1 µg/100 µL	AATTAGCCGTCGAGA
900009786	C5b-9 (MAC)	Mouse IgG2a κ	TotalSeq-B5101/AF488	1 µg/100 µL	TACTCACCGTAGTAC
359139	CD195	Rat IgG2b κ	TotalSeq-B0141	1 µg/100 µL	CCAAAGTAAGAGCCA
304066	Isotype Control	Mouse IgG1 κ	TotalSeq-B0090	1 µg/100 µL	GCCGGACGACATTAA
400185	Isotype Control	Mouse IgG2a κ	TotalSeq-B0091	1 µg/100 µL	CTCCTACCTAAACTG
400689	Isotype Control	Rat IgG2b κ	TotalSeq-B0095	1 µg/100 µL	GATTCTTGACGACCT

Supplemental Table 2:
Labeling Molecules for Immunohistochemistry (Choroidal sections):

Manufacturer	Labeling Molecule	Molecule Type	Host Species	Labeling Concentration	Tag
Abcam	Anti-C5b-9	Antibody	Rabbit	16.67 μg/mL	N/A
Novus Biologicals	Anti-C5b-9	Antibody	Mouse	3.33 μg/mL	N/A
Millipore-Sigma	Anti- α-smooth muscle actin	Antibody	Mouse	3.75 μg/mL	Cy3
Thermo Fisher	Anti-rabbit	Antibody	Donkey	10 μg/mL	AF488
Thermo Fisher	Anti-mouse	Antibody	Donkey	10 μg/mL	AF488
Vector Laboratories	UEA-I	Lectin	*Ulex europaeus*	20 μg/mL	Biotin
Vector Laboratories	Texas Red	Fluorophore	N/A	10 μg/mL	Streptavidin

Supplemental Table 3:
Labeling Molecules for Immunohistochemistry (Choroidal whole mounts):

Manufacturer	Labeling Molecule	Molecule Type	Host Species	Labeling Concentration	Tag
Abcam	Anti-C5b-9	Antibody	Rabbit	16.667 μg/mL	N/A
Millipore-Sigma	Anti- α-smooth muscle actin	Antibody	Mouse	3.75 μg/mL	Cy3
Thermo Fisher	Anti-rabbit	Antibody	Donkey	10 μg/mL	AF488
Vector Laboratories	UEA-I	Lectin	*Ulex europaeus*	20 μg/mL	Biotin
Vector Laboratories	DyLight 649	Fluorophore	N/A	10 μg/mL	Streptavidin

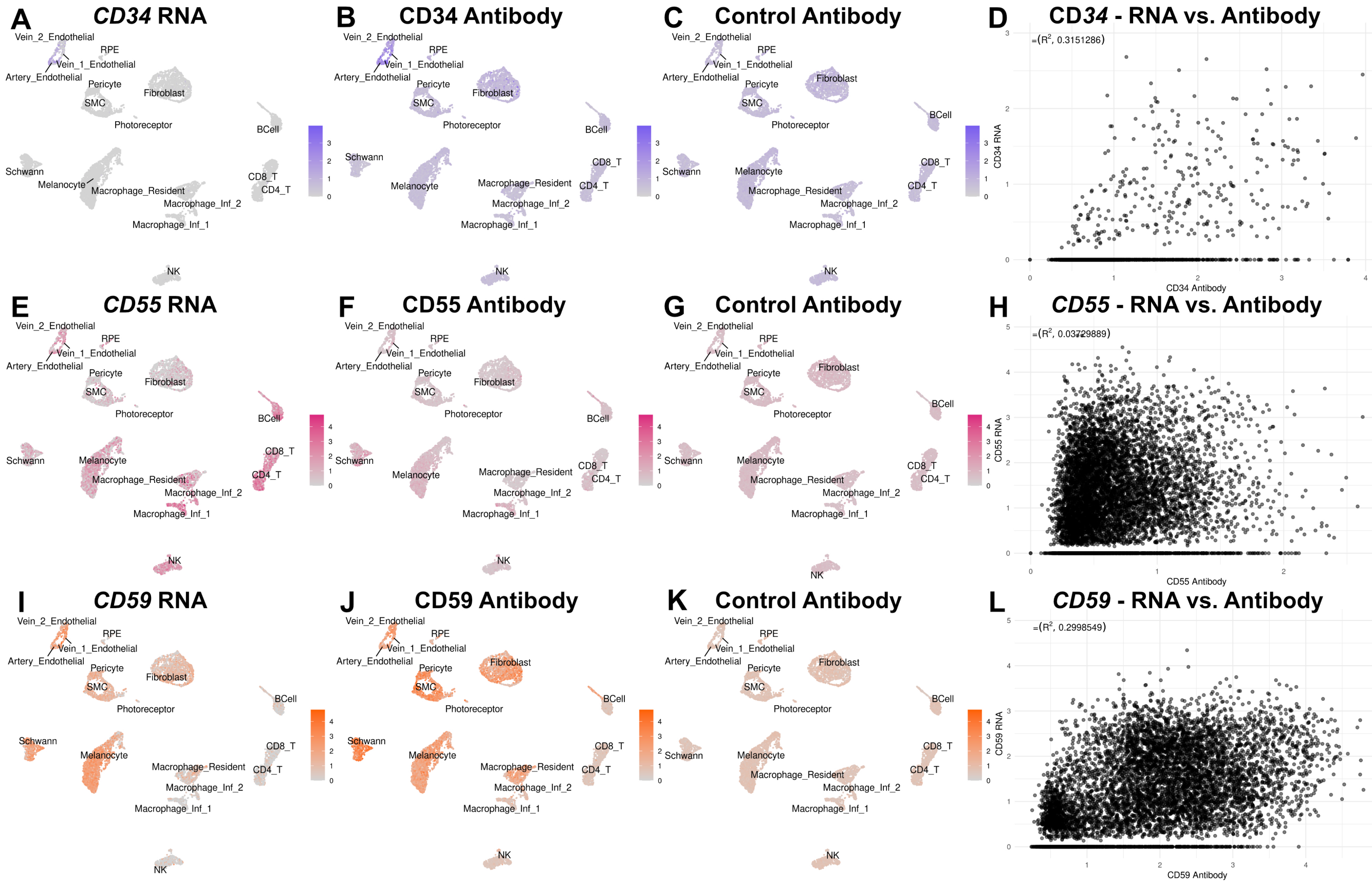

Supplemental Figure 1. CD34, CD55, CD59 surface detection across all cells. Gene expression plots for CD34, CD55, and CD59 are shown in (A), (E), and (I), respectively. Corresponding antibody signals are shown in (B), (F), and (J). Isotype control signal is shown in (C), (G), and (K). Gene expression vs. antibody signal per cell is plotted across all cells for *CD34*, *CD59*, and *CD55* in (D), (H), and (L), respectively.

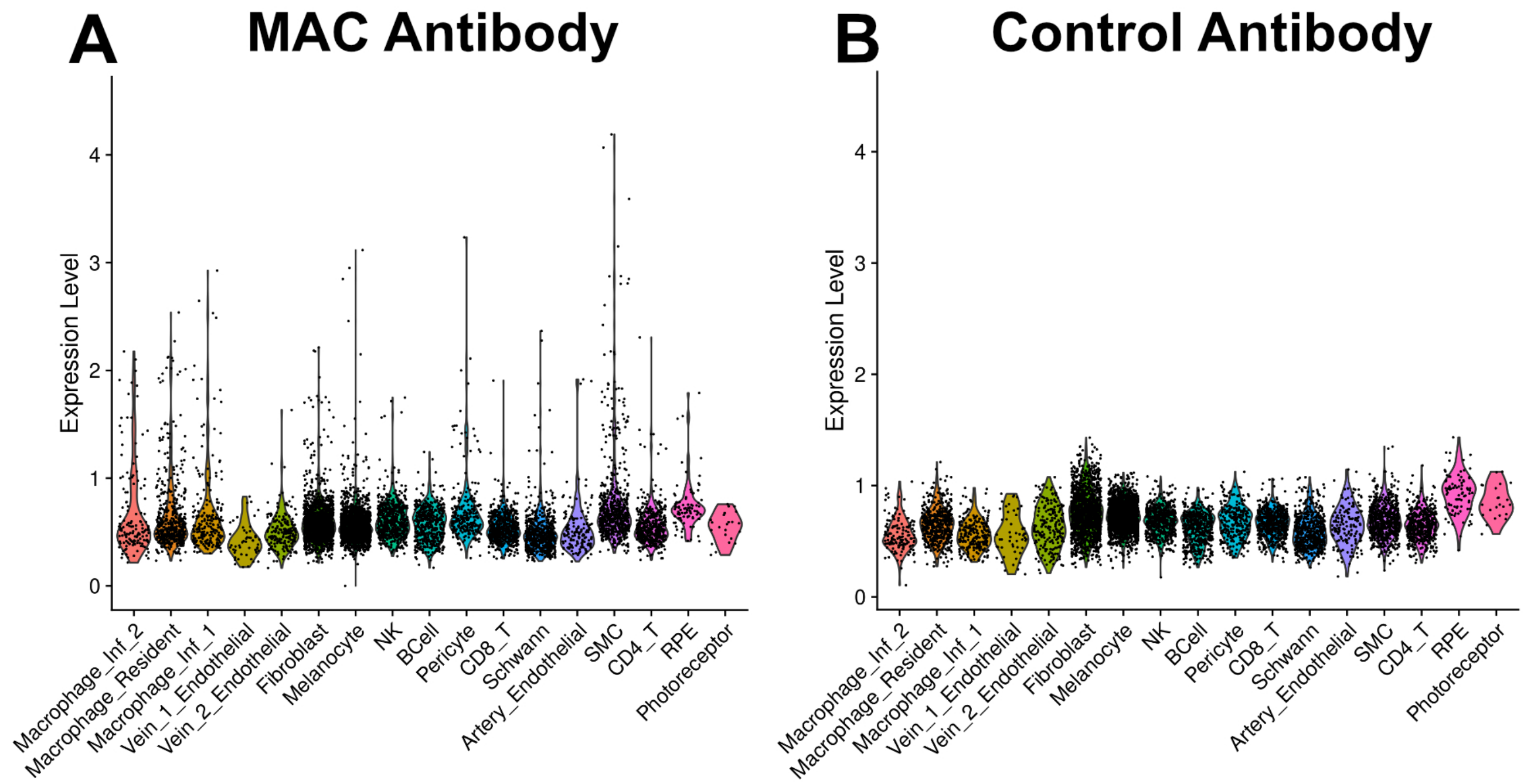

Supplemental Figure 2. MAC detection across all cell types. The MAC antibody signal across our recovered cell types is plotted in (A). The signal for the corresponding isotype control (IgG2a) in each cell type is shown in (B).

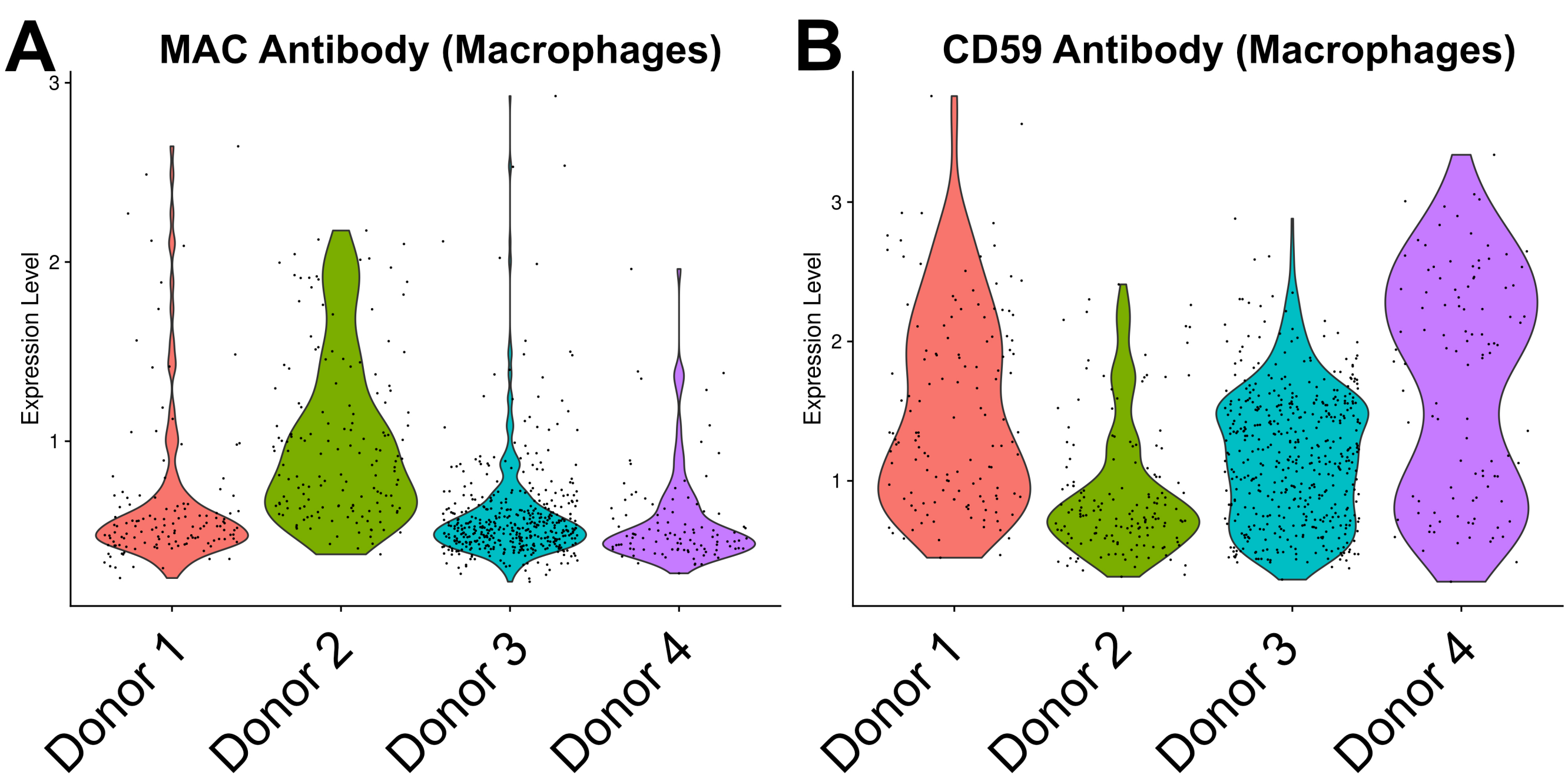

Supplemental Figure 3. MAC on macrophages across donors. MAC antibody detection on the macrophage family is plotted across all donors in (A). CD59 antibody detection is shown in (B) across the same donors.

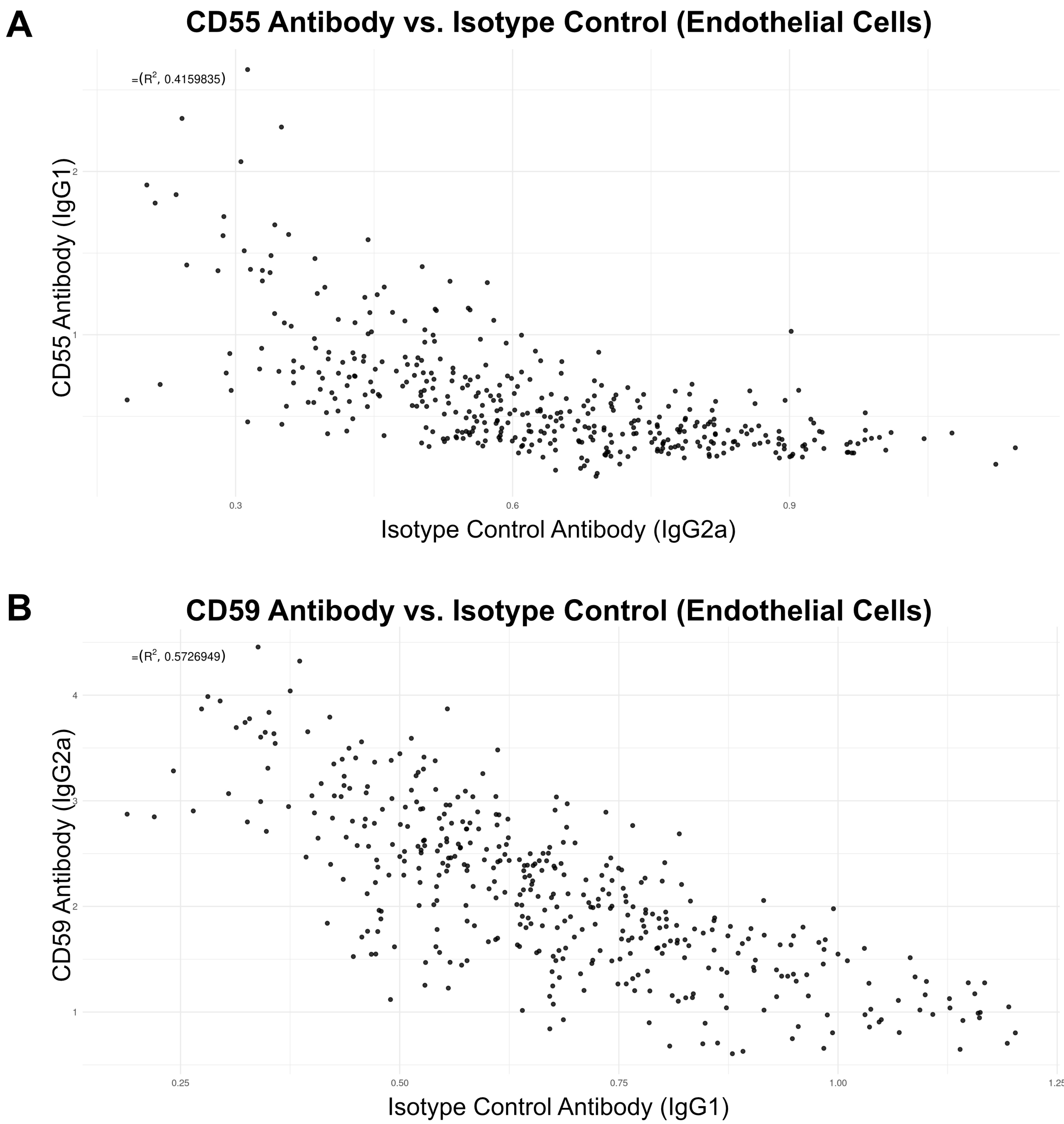

Supplemental Figure 4. Complement regulators vs. isotype controls on endothelial cells. A scatterplot of CD55 antibody (IgG1) detection vs. the CD59 antibody's isotype control (IgG2a) on a per-cell basis is shown in (A). A similar plot of CD59 antibody (IgG2a) detection vs. the CD55 antibody's isotype control (IgG1) is shown in (B).

A

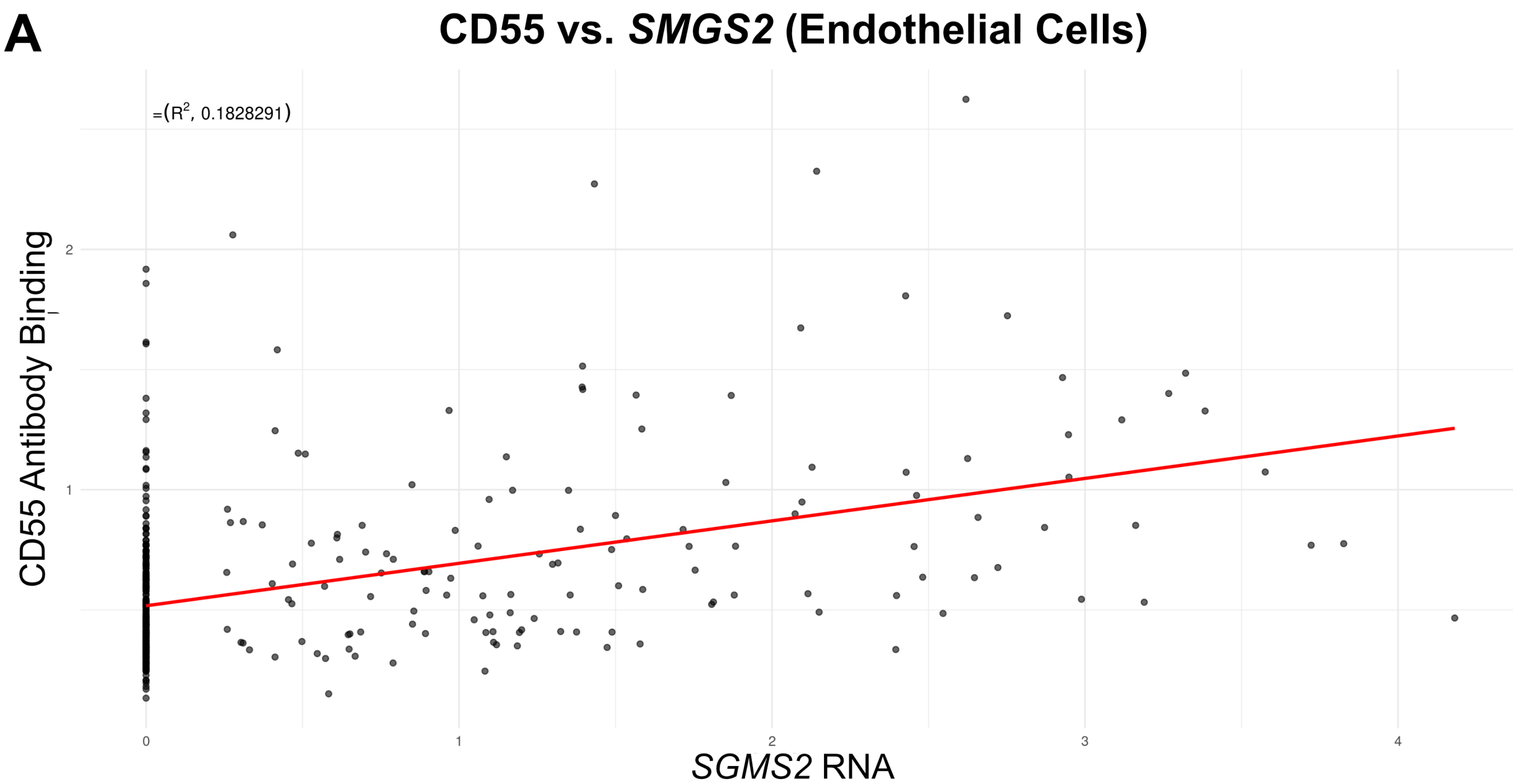

B

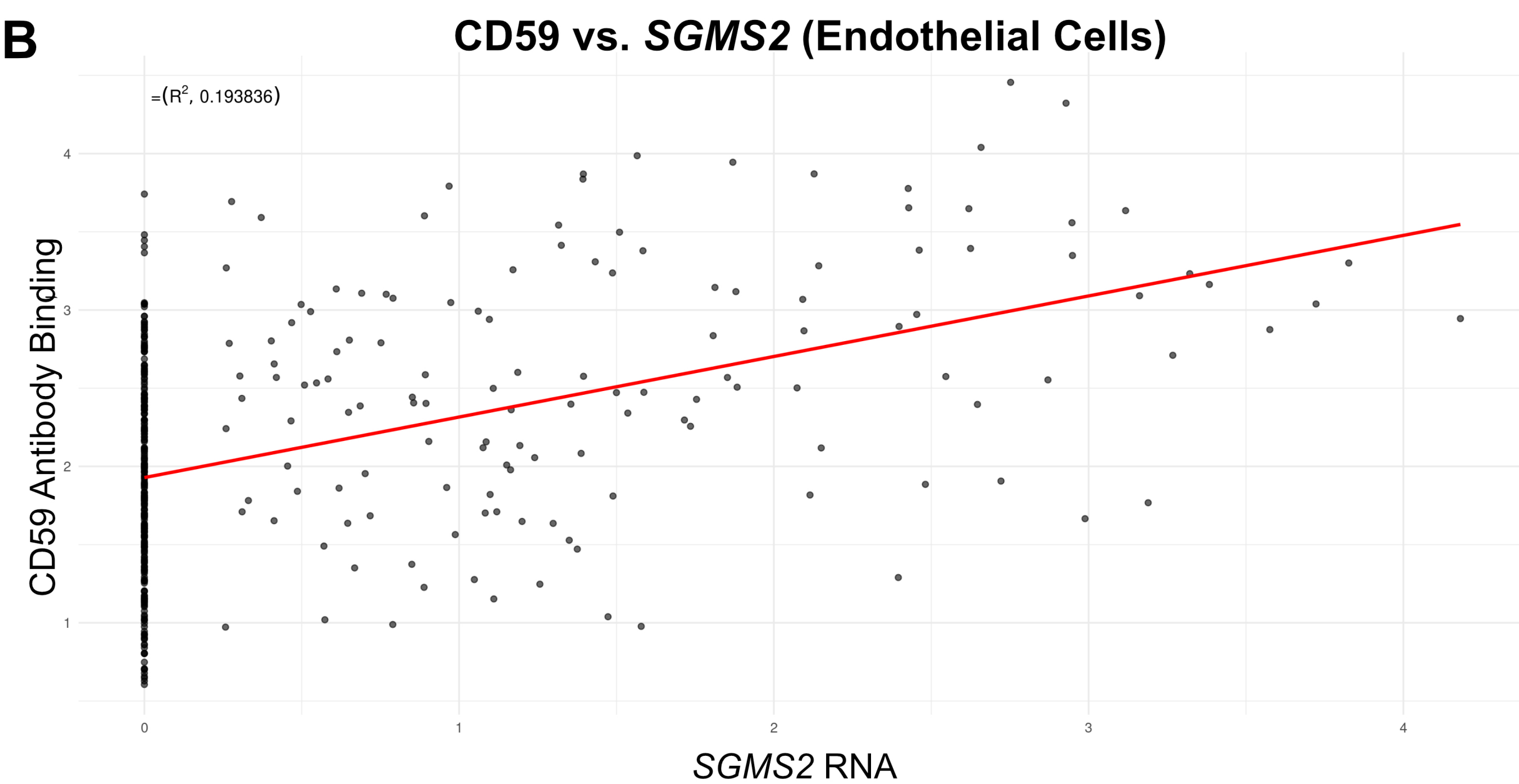

Supplemental Figure 5. Complement regulator detection vs. *SGMS2* expression on endothelial cells. A scatterplot showing CD55 antibody detection vs. *SGMS2* gene expression on a per-cell basis is shown in (A), while the same plot for CD59 is shown in (B).
